# Supplementary material for: Reevaluating the relationship between EGL-43 (EVI1) and LIN-12 (Notch) during C. elegans anchor cell invasion
Source: Biol Open. 2022 Dec 6;11(12):bio059668. doi: 10.1242/bio.059668 (PMC9751802; doi:10.1242/bio.059668)
Supplement: Supplementary information [file biolopen-11-059668-s1.pdf]

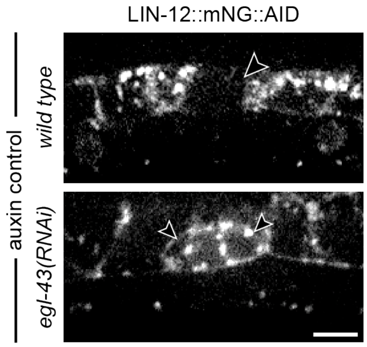

Fig. S1. EGL-43 depletion leads to ectopic LIN-12 expression on the AC membrane. Micrographs of mid-L3 larvae at the time of AC invasion expressing LIN-12::mNeonGreen::AID in wild type (top; extracted from Fig. 3A) or after *egl-43(RNAi)* (bottom; extracted from Fig. 3B) in the absence of 5-Ph-IAA. Treatments were initiated at the L1 larval stage. Note the presence of nuclear LIN-12::mNeonGreen::AID in the underlying D cells of wild-type controls. Scale bar, 5 µm.

Table S1. **Strains**

Name	Genotype	Source
DQM1125	*bmd284 I*; *ljf33 III*; *bmd299 V*	This paper
DQM1159	*bmd284 I*; *ieSi58 IV*	This paper
DQM1233	*bmd284 I*; *wy1514 II*; *bmd202 V*; *qy20 X*	This paper

Biology Open • Supplementary information

Table S2. Primers

Name	Sequence (5' – 3')	Type	Amplicon	Template
DQM1136	tgtaaaacgacggccagtgcggccgcGT TTGTGCAACAAATTGAGGAAG	Forward	rpl-28p::TIR1(F79G)::T2A	pCMH2123
DQM1137	caggtgacgtcgttggtcatgggcccTCC TGGGCCAGGATTCTC	Reverse	rpl-28p::TIR1(F79G)::T2A	pCMH2123
CY419	caataagacacgcgcgccgcccatccctc gtgcatcttccctttggggtgtgtcacaggg ctcattcttgtgacgtgcgcttcccaccttt aAcagtgtgttatcaatctccgtcttattca ttagtgaataaatattccaggacggttgga cgtgcccaacgtgtcaaagtcagGGAG CATCGGGAGCCTCAGGAGCat	Forward	egl-43::TagRFP-T::AID::egl-43	pWZ203
CY420	agttttcaaaataataactaacctgtgttg ccatttgaagtatatgtggccaatatggca cggaacctaatccacctgctccgctcaatc cggcaagttgcgccatcaatggtttgtaga gtgcagtcatctcgcaaactggtcgatgct tcgttagtgccgcttgtgatggcatGGCT CCGCTAGCTCCTGACTTGACG	Reverse	egl-43::TagRFP-T::AID::egl-43	pWZ203

Table S3. Plasmids

Name	Backbone	Description
pWZ259	pWZ192	rpl-28p::TIR1(F79G)::T2A::DHB::2xmKate2

Table S4. Guides

Locus	Sequence (5' – 3')	Description
egl-43	GACGGAGATTGATAACACAC	Located upstream of exon 6

Biology Open • Supplementary information
